# The Transactions of the American Medical Association, Instituted 1847

**Published:** 1851-01

**Authors:** 


### EDITORIAL DEPARTMENT.

*The Transactions of the American Medical Association, Instituted* 1347. Vol. 111. Philadelphia, 1850. pp. 499.

The volume of transactions of the American Medical Aassociation, for the present year, is less in bulk, by nearly one half, than that for 1849. It is not to be inferred from this fact, that, as respects the progress of the medical sciences in America, the past year bears a corresponding proportion to the year preceding, nor that the volume for the present year is in value one half less than its immediate predecessor. The diminution is chiefly owing to the reports of standing committees having been restricted in their scope to American Medical Science, but, in some measure, also, to greater condensation in their preparation. There is less diffusiveness, and less irrelevant and appended matter in this volume, when compared with that for 1849; and, hence, it is really more valuable, and will, we doubt not, be more acceptable to the majority of readers. By this remark we would not be understood to imply any invidious comparisons respecting the ability displayed in the reports of 1850, as contrasted with those of 1849. The latter were, perhaps, quite as creditable to their authors as are the former. The improvement consists in greater brevity, and in an exclusive nationality. The latter quality is desirable for the sake of consistency, at least, in the transactions of an *American* Association.

*The Report of the Committee on Medical Science, comes first in order.* Dr. Usher Parsons, of Providence, R. I., Chairman.

This extends over fifty pages. It is a succinct, clear, and candid exposition of the progress of Anatomy, Physiology, General Pathology, and Therapeutics, etc., during the year; being just what should have been expected from the able and judicious Chairman of the Committee.

*The Report on Practical Medicine and Epidemics follows, and occupies thirty-eight pages.* Prof. I. K. Mitchell, Chairman.

Nearly the whole of this Report is devoted to Cholera, which, as the reader will recollect, was not only the chiefly absorbing topic of Medical Leterature, during the year, but the absorbing disease in another sense, i. e., all other epidemics, and, to a certain extent, sporadic affections, having been merged into this epidemic. The report contains much interesting and valuable statistical information, mainly derived, however, from the city of Philadelphia. It is to be regretted that the observations and statistics in different sections, which had been communicated during the year in the different Medical Journals, had not been more fully embraced in the Repot, so that the transactions would have embodied, more completely, all the available facts appertaining to the prevalence of the Epidemic.

*The Report of the Committee on Medical Education*, Prof. JOSEPH ROBY, Chairman, is brief, occupying only eight pages.

We have given this Report in full, in the Electic department of the Dec. No., 1850, of our Journal. In our judgment, it presents an able and candid statement of the position of the subject of Medical Education, as it stands before the Association, and the medical public at the present time. The views of the author seem to us to do justice to all the parties interested in the discussion of the subject—the Association—the Medical Schools—the Profession at large, and medical students. This was the impression received when we listened to the reading of the Report, and it is confirmed by its perusal. We have the less reluctance to express such an opinion, beeause the Report, and the conduct of its author, were, as it seems to us, harshly and unfairly treated by some members of the Association. The ground of complaint against the report appeared to be that it was not in strict accordance with the character of previous reports, and action of the Association; as if, forsooth, a Chairman of a committee is to be instructed by the opinions of his predecessors, as to the views he is to present; or, as if the Association, at its successive annual meetings, is bound to an uniform tenor of action! These positions, preposterous as they must seem to most intelligent persons, appear to be seriously assumed by some and made the basis of opposition to new propositions for legislative deliberation. The cavilings to which we have referred, were the more unbecoming, because the Chairman of the Committee was not present to defend himself, or to make explanations; and, as it appears, points which were made the occasion of personal invective, were susceptible of complete and satisfactory explanation.

*Report of the Committee on Medical Literature.* ALFRED STILLE, M. D., Chairman.

This report occupies *fifty* pages. The preparation of a report on Medical Literature is a delicate duty, involving, as it does, an impartial discrimination, which, it may well be imgined, cannot easily be exercised without danger of giving offence to some, and, unless managed with tact, may wound the feelings of the literary aspirant, in an uncalled for degree. The Report by Dr. Stille, is executed with his usual ability and elegance, and we believe it is not open to censure on the score of either candor or fairness.

Appended is a list of all the original articles, of interest, published in American Journals during the year, arranged according to their subjects. This will prove useful for reference, and we hope the plan will be followed by the authors of subsequent Reports on Medical Literature.

*Report of a Special Committee, on the means of Encouraging a National Medical Literature.* Prof. W. E. HORNER, Chairman.

The following resolution embodies the conclusions at which this Committee arrived:—*Resolved,* That in the opinion of this Association, the only legitimate means within our reach, for the encouragement and maintenance of a National Medical Literature, are to increase the standard of preliminary and professional education, required of those who would enter the medical profession; to promote the circulation, among the members of the profession, of the medical journals of the day; to encourage the establishment of District Medical Libraries, and to induce every practitioner to cultivate, with care, the fields of observation and research, that are within his reach.

These several *means* are, of course, very desirable in themselves, but they would be the legitimate *ends* of a better encouragement of a National Medical Literature.

*Report of a Committee on a Memorial to Congress, in favor of an International Copy-right Law.* Prof. GEO. B. WOOD and Dr. ISAAC HAYS, Committee.

The Committee reported a Memorial, which was adopted by the Association, without debate. This we regretted, not because we are prepared to deny the policy of the measure, but we opine that the question has not been sufficiently considered by the medical profession to take action respecting it. Some members of the Profession, as we know, who have bestowed upon the subject considerable reflection, have been led to doubt the expediency of petitioning for a copy-right law. Moreover, as a matter for Congressional action, the subject embraces a wider scope than medical publications, and involves other considerations than those relating to Medical Literature. Hence, it is not to be expected that paramount importance will be attached to petitions from the Medical Profession, and we would reserve our influence with the National Legislature, for occasions in which we should have more certainty of effecting something, and for objects more clearly to be desired.

*Report of the Committee on Publication.* ISAAC HAYS, M. D., Chairman, and Report by the Treasurer, Dr. HAYS.

The Committee on Publication reported the following resolutions, which were adopted:—

1st. *Resolved,* That the assessment for the present year shall be three dollars.

2d. *Resolved,* That those delegates who pay the assessment shall be entitled to one copy of the Transactions of the present year, and that the payment of two dollars in addition shall entitle them to two additional copies.

3d. *Resolved,* That permanent members shall be entitled to one copy of the Transactions of the present year on the payment of two dollars, and three copies on the payment of five dollars.

4th. *Resolved,* That societies which are represented at this meeting shall be entitled to copies for their members on the same terms that such copies are furnished to permanent members.

5th. *Resolved,* That the permanent members, unless present at the meeting as delegates, shall not be subject to any assessment.

6th. *Resolved,* That any delegate who is in arrears for his annual assessment, shall not be considered as a permanent member.

7th. *Resolved,* That the several committees be requested to bring to the meeting of the Association their reports correctly and legibly transcribed; and that they be required to hand them to the Secretaries as soon as they have been read."

The Treasurer's Report exhibits a balance in the Treasury of seventy-three dollars and fifty-two and a half cents.

*Report of the Committee on Public Hygiene.* Prof. JOSEPH M. SMITH, Chairman.

The report proper embraces *twenty-three* pages, and is devoted mainly to the consideration of the source of the poison producing Typhus Fever, which the author thinks is produced by excretions from the living human body, and more especially the animal matter entering into the composition of the cutaneous and pulmonary exhalations. In other words he refers the origin of Typhus to what has been distinguished as *idio miasmia*

The considerations which are adduced in support of this view, or rather in elucidation of it, are interesting, and striking. The subject is handled with much ability.

Appended to this report, are two papers, one on the *Sanitary Condition of Massachusetts aud New England*, by Dr. Edward Jarvis ; the other on the *Hygienic Charactaristics of New Orleans*, by Dr. J. C. Simons. Both papers will repay a careful perusal, and will serve to direct attention to important questions, in which is the subject of public health—a subject which has secured a degree of attention inversely in proportion to its magnitude and importance.

*Report on Adulterated Drugs, Medicines, Chemicals, etc.* Prof. R. M. Huston, Chairman. 18 *pages.*

Nothing but want of space, together with the expectation that the Transactions will circulate extensively among members of the profession, restrains us from inserting the greater portion of this report, so valuable are the facts which it embraces, and so particularly important is the subject to the practitioner. The evils arising from adulterations, and the sale of spurious medicines, are not generally half enough appreciated. These evils do not relate solely to the cure of disease in the cases in which unreliable articles are dispensed; but the therapeutical experience based on them must necessarily become vitiated. In the latter point of view, the injury can hardly be estimated; it must be very great. The committee recommended the following resolutions, which were adopted :—

"1st. *Resolved,* That the various State and local medical sooieties be requested annually to appoint boards of examiners, whose duty it shall be to procure specimens of drugs from the stores within their limits, for examination, and report upon the same to thcir respective societies, at least once in every year.

2ndly. *Resolved,* That the respectable druggists and apothecaries throughout the United States be requested to take active measures for suppressing the fabrication and sale of inferior and adulterated drugs, and that it be respectfully suggested to them, whenever practicable, to form themselves into societies or colleges for the promotion of pharmaceutical knowledge and general improvement in their profession.

3dly. *Resolved,* That a committee be appointed, consisting of one member from each state here represented, whose duty it shall be to collect information in regard to adulterated and spurious drugs, and report the same at the next meeting of the Association."

*Report of the Committee on Indigenous Medical Botany.* Prof. Eli Ives, Chairman.

This report is quite brief, and is confined to an account of the personal experience of the Chairman, and of Dr. Benett, of S. C., on the virtues of a few plants not before enumerated, or the medicinal effects of which have been only partially described.

*Report of the Standing Committee on Surgery.* Prof. R. B. Mussey, Chairman.

This report should have followed that on Practical Medicine, but was not inserted in its proper place in consequence of its not having been received by the Committee until after the foregoing reports were in type. The report extends over *sixty-four* pages, and, so far as we may judge, contains a faithful statement of improvements, etc., in the department of Surgery, during the year. Appended to the report are the following papers :—*On the use of Anaesthetics*, by J. D. Warren, M. D. *On the same subject*, by W. L. Atlee, M. D. *On the use of Chloroform*, by J. D. Gross, M. D. *On the Result of Operations for the cure of Cancers*, by J. C. Warren, M. D.

The Report on Surgery, with the papers appended, closes the volume of Transactions proper. In an appendix, are embraced several papers, as follows :

1. Protest of the delegates from the state of Alabama, against statements reflecting on the character of the medical profession of that state, contained in the Report on Medical Education, for the year 1849.

2. A discussion of the question, has the Cerebellum any special connection with the sexual propensity, or function of generation? By Prof. .N S. Davis.

3. The Obstetrical Extractor, by Prof. John Evans.

4. A brief notice of some of the Physicians of the United States, who have died within a few years. By Stephen W. Williams, M. D.

In connection with the Transactions, we have, in this volume, the official publication of the minutes of the third annual meeting of the Association. We observe in these a striking discrepancy with the account given by us in a former number of this Journal, relating to the action taken on the subject of Medical Education. We reserve this matter for further notice in a subsequent No.

---

The foregoing was in type for our last No., but excluded for want of space. We will now indicate the discrepancy in the published minutes, to which allusion is made in the last paragraph of the preceding article.

On page 35, we find as follows:—"The Chairman of the committee of the whole reported, that they had had under consideration the preamble and resolutions of Dr. Blatchford, of N. Y., and certain other resolutions, herewith submitted, proposed by Drs. Lawson & Drake, of O., Gross, of K. Y., and Theobold, of M. D., which were recommended by resolution of Dr. Flint, of N. Y., to be referred to the standing committee on education for 1851: *and that they afterwards adopted the accompaning resolution of Dr. Caspar Morris, of Pa., offered as a substitute for the above.*" We have italicised the portion to which the error specially relates. The following quotation from page 36, contains the resolution of Dr. Morris, refered to in the foregoing extract, and reiterates the above statement: "Resolution offered by Dr. Morris, of Pennsylvania, as a substitute, passed in committee of the whole, *reported to the Association, and adopted*:

*Resolved*, That the recommendations of this Association, at its former meeting, in regard to Medical Education, be re-affirmed, and that private preceptors be still urged to receive into their offices only those duly qualified by previous education to engage in the study of medicine."

Now, had we not participated in the proceedings at this stage, (having offered the resolution of reference to the standing committee on educations for 1851,) we might distrust the accuracy of our recollection, but we think we can hardly be mistaken in the opinion that the above account is erroneous. Moreover, we think that the account itself, as it stands in the published minutes, shows that there is an error, in other words, that the course of proceedings, as given, is inconsistent and unparliamentary. We hasten to remark that we would not, for a moment, be thought to imply that there is any *intentional* error in the record by the Secretaries of the Association. The error, (if error there be,) doubtless arose from some confusion in the proceedings at the period to which it relates.

On the second day of the session of the Association, May 8th, after the reading of the Report by the Chairman of the Committee on Medical Education, a series of Resolutions was submitted by Dr. Blatchford, (a member of the Committee,) as a minority Report. The discussion of these resolutions, proposed amendments, substitutes, etc., occupied the Association during the whole of that day, and on the morning following the Association resolved itself into a Committee of the Whole to continue the discussion, Dr. Knight, of New Haven, being Chairman of the Committee. The discussion in Committee of the Whole continued during the morning session until a motion was made by the writer that the *Committee rise and report a re-*

*commendation* to the Association to refer the whole subject to the standing Committee on Education for 1851. The motion prevailed by a large majority. Dr. Lopez, Vice President, resumed the Chair, and the Report was made at once by Dr. Knight, Chairman of the Committee. These being the facts, the error consists in the statement that Dr. Morris' resolution was passed in Committee of the whole *subsequently* to the motion by the writer. That resolution, as we recollect distinctly, was offered prior to the motion referred to, and of course, by this motion was referred, as well as the other resolutions mentioned, to the standing Committee on Education for 1851.

The reader conversant with the rules of parliamentary usage will perceive that the facts, as we have stated them, must be correct. After a motion has prevailed to rise and report, in Committee of the Whole, no resolution can be adopted without a re-consideration of the motion to rise and report. It is in no wise probable that such a blunder could occur with Dr. Knight, (an admirable presiding officer,) in the chair, and with many members present who were practically familiar with the laws regulating deliberative bodies.

The *inconsistency* of the proceedings, as they are published, shows, equally, that they must contain the error pointed out. As they read, the resolutions of Drs.Blatchford, Lawson, Drake, etc., were recommended, by resolution of Dr. Flint, to the standing Committee on Education for 1851, but that afterward was adopted a resolution by Dr. Morris *as a substitute for the above resolutions.* To make a reference of certain resolutions to a committee, and then directly to adopt a resolution as a substitute for the resolutions referred, without reconsidering the motion of reference, would be absurd.

According to the proceedings as published, the Association again committed itself to its previous recommendations on the subject of Medical Education, but if these proceedings do contain the error to which we have adverted, the action of the Association on the subject of Medical Education was negative, and the whole matter is referred to the next Medical Congress. The point, thus, it will be perceived is not altogether trivial.

In making this criticism on the published proceedings, it is due to ourselves to add that we are not opposed to the recommendations of the Association for the re-affirmation of which Dr. Morris' resolution provided. These recommendations relate mainly to increased duration of instruction in Medical Colleges. We are in favor of this measure, and are ready to advocate it to the best of our ability. As respects the mode of increasing instruction advised by the Association, viz., lengthening the term to six months, we are not so clear that this is the best that can be desired. But we reserve remarks on this topic for another time.

